# Linking the structure of vascular bundles and mineral element deposition reveals the hub role of nodes in bamboo

**DOI:** 10.1093/hr/uhaf113

**Published:** 2025-04-24

**Authors:** Xianyu Pan, Ji Feng Shao

**Affiliations:** National Key Laboratory for Development and Utilization of Forest Food Resources, Zhejiang Agriculture & Forestry University, No. 666, Wusu Road, Lin’an, Hangzhou 311300, China; Bamboo Industry Institute, Zhejiang Agriculture & Forestry University, No. 666, Wusu Road, Lin’an, Hangzhou 311300, China; National Key Laboratory for Development and Utilization of Forest Food Resources, Zhejiang Agriculture & Forestry University, No. 666, Wusu Road, Lin’an, Hangzhou 311300, China; Bamboo Industry Institute, Zhejiang Agriculture & Forestry University, No. 666, Wusu Road, Lin’an, Hangzhou 311300, China

## Abstract

Nodes are a distinct feature of bamboo plants, categorized into three main types: culm, shoot, and rhizome nodes. However, the latter two are often overlooked due to their underground growth, resulting in a limited understanding of their structure and function. In this study, we examined the structure and mineral elements deposition in the nodes of Moso bamboo (*Phyllostachys edulis*). Our findings indicate that all three node types possess a complex yet well-organized vascular bundle system, with notable differences. Culm nodes feature enlarged vascular bundles with distinct xylem and phloem regions, whereas shoot and rhizome nodes have less developed phloem regions. The rhizome node contains a vascular structure of crown root and coronary shoot bud, which is absent in culm and shoot nodes. In the culm node, iron accumulation decreases gradually from the bottom to the top, primarily localizing in cells near the enlarged and small vascular bundles. Zinc is deposited in both the enlarged and small vascular bundles in the lower part of the node. In contrast, calcium accumulates predominantly in the upper part, particularly in cells adjacent to enlarged and small vascular bundles including diffuse and parenchyma cells. Potassium is distributed throughout most cells but is less abundant in the pith cavity and xylem transfer cells. In shoot and rhizome nodes, iron, zinc, calcium, and potassium exhibit specific regional and cellular deposition patterns. Overall, the vascular structure and mineral element deposition patterns suggest that bamboo nodes function not only as tissue junctions but also as critical hubs for mineral element deposition and distribution.

## Introduction

Vascular plants have a fundamental structural unit known as the phytomer, which serves as the basis for their architecture and growth [1, 2]. In trees, a typical phytomer generally consists of a node and an internode accompanied by one or more leaves, although the node may not always be readily apparent [1]. Conversely, in some herbaceous plants, particularly in the culms of Gramineae family members such as rice, wheat, maize, and barley, nodes are more prominent and easily identifiable [3, 4]. In common grasses, nodes function as crucial interfaces between the leaves and stems [2], while in Gramineae plants, they act as junctions connecting leaves, branches, tillers, and roots.

Bamboo, a member of the Gramineae family, and the Bambusoideae subfamily, is widely distributed across tropical and subtropical regions. A typical bamboo plant is characterized by an upright tubular culm, segmented by nodes, which are distinguished by an internal diaphragm, a sheath scar, and a nodal ridge [5]. As a unique structural feature, nodes play a key role in differentiating bamboo from other plants. Over the past few decades, extensive studies have functionally characterized bamboo nodes, focusing on their mechanical properties and structural composition. Detailed investigations have explored the physical and mechanical attributes of bamboo nodes, including tensile strength, compressive strength, bending strength, and shear strength [6–10]. These studies suggest that the nodal region of bamboo may represent a structurally weaker point, exhibiting lower strength compared to internodal sections [10]. However, from a broader perspective of the entire bamboo culm, nodes are believed to enhance stiffness and stability, acting as spring-like joints that provide support against bending forces [11–13]. This structural role is further reinforced by the complex three-dimensional woven arrangement of vascular bundles and the hierarchical fiber reinforcement schemes observed in bamboo nodes [10, 14].

In addition to mechanical properties, the anatomical structure and bundle organization of bamboo nodes have also been extensively studied. Early research relied on two-dimensional imaging techniques, such as optical microscopy and scanning electron microscopy, to reconstruct the structure of bamboo nodes [15, 16]. More recently, X-ray microtomography has emerged as a powerful tool for investigating the intricate vascular systems within bamboo nodes [17]. Using this technique, researchers have successfully reconstructed a three-dimensional visualization of *Bambusa emeiensis* bamboo nodes, revealing the orientation of axial and transverse vascular bundles and their interconnections. These studies have identified parenchyma as the dominant tissue, constituting 65.7% of the node’s composition, followed by fibers (30.5%) and conducting tissue (3.8%) [18].

Despite these advancements, most studies have primarily focused on nodes within the culm. For instance, three-dimensional reconstructions of bamboo nodes have been conducted for species such as *Phyllostachys edulis*, *B. emeiensis*, *Pleioblastus amarus*, *Bambusa rigida*, *Pleioblastus gozadakensis* Nakai, *Qiongzhuea tumidinoda* [5, 18, 19]. However, these studies largely concentrate on the culm node while overlooking the integral role of nodes in connecting leaves, branches, and tillers. As previously mentioned, nodes function not only as a junction between internodes but also as key connector between different plant structures. Therefore, to gain a more comprehensive understanding of the vascular bundle system and the functional significance of nodes, a holistic and integrated study of encompassing branches, leaf sheaths, and other associated structures is essential.

**Figure 1:**
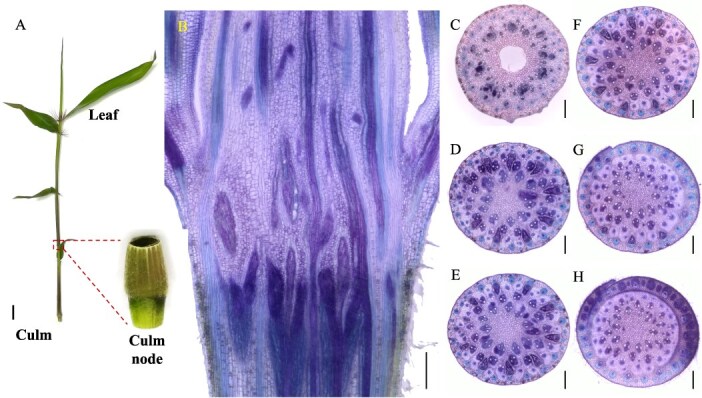
Morphology and vascular systems of the bamboo culm node. (A) Morphology of the bamboo culm node. (B) Longitudinal view of vascular systems in the bamboo culm node. (C–H) Transverse view of vascular systems in the bamboo culm node from lower to upper in six positions. Transverse and longitudinal sections (100 μm) of Moso bamboo nodes were cut with a microtome and double stained with toluidine blue and safranine. Longitudinal view (B) of the whole node and transverse view (C–H) of different sections from lower to upper nodes were photographed. Scale bar in A is 1 cm, and scale bars in B–H are 300 μm.

In addition to culm nodes, bamboo has two other types of nodes that are often overlooked during growth: the shoot node and the rhizome node. The shoot node, located on developing bamboo shoots, is wrapped by a sheath, and typically consists of a sheath scar and a diaphragm, forming the junction between the sheath and the internode. In contrast, the rhizome node, which develops underground, connects roots, shoot buds, and internodes. Despite their importance, the anatomical structure and vascular bundle arrangement of nodes in shoots and rhizomes remain poorly understood.

Limited research has been conducted on shoot and rhizome nodes, partly due to the challenges associated with sampling these underground structures and obtaining comprehensive images of their complex anatomy. To address this limitation, a smaller, more easily sampled model is needed for experimental investigations. In this study, we utilized Moso bamboo seeds (*P. edulis*), a bamboo species with widespread distribution, to cultivate bamboo seedlings for node collection in a hydroponic system. Using this miniature node model, we successfully visualized the vascular structure of culm, shoot, and rhizome nodes. Additionally, we investigated the role of nodes as hubs for mineral elements distribution by analyzing mineral deposition patterns within these structures.

## Results

### Anatomy and vascular bundles structure of the culm node

In bamboo seedlings, nodes are enclosed by a leaf sheath (Fig. 1A), and each node is connected to adjacent nodes through an internode (Fig. 1A). In a longitudinal view, the vascular bundles within the node exhibit a complex arrangement, consisting of both axial and transverse vascular bundles that traverse the nodal region (Fig. 1B). The axial vascular bundles become denser and more intricate in the lower part of the node, while they appear simpler and less dense toward the top. This gradient is particularly evident in the transverse view (Fig. 1C–H). Some vascular bundles undergo structural modifications as they transition from the internode to the node, developing into enlarged vascular bundles primarily composed of a fibrous sheath, xylem, and phloem (Fig. 1C and D). These enlarged vascular bundles gradually diminish in size from the low to the upper region of the node, forming a radial gradient (Fig. 1E–G). At the top of the node, certain vascular bundles differentiate into leaf sheath vascular bundles that encircle the nodal region (Fig. 1H).

In the typical nodal region (Fig. 1D), vascular bundles differentiate into three distinct types: enlarged vascular bundles, diffuse vascular bundles, and transit vascular bundles. Each type can be further classified as either large or small (Fig. 8A). The enlarged vascular bundles (large E^L^VB and small E^S^VB, shown in red and purple, respectively, in Fig. 8) are alternately distributed in a radial gradient along the outer region of the node. The larger E^L^VB looks like an irregular isosceles triangle (occupying approximately one-third of the node’s width), while the E^S^VB exhibits an oval shape (Fig. 8A).

Diffuse vascular bundles, including large D^L^VBs (green) and small D^S^VBs (pink), are distributed around the enlarged vascular bundles (Figs 1D and 8A). Meanwhile, transit vascular bundles, consisting of large T^L^VBs (yellow) and small T^S^VBs (orange), are located in the innermost and outermost layers, respectively (Figs 1D and 8A). As the node extends vertically, the T^L^VB maintains a consistent shape, whereas the T^S^VB splits into two vascular bundles near the D^L^VB and ultimately positions itself between the D^S^VBs, which continue to grow within the leaf sheath. These vascular bundles are horizontally interconnected through ‘nodal vascular anastomosis’ facilitated by bundle sheath cells and parenchyma cells.

### Anatomy and vascular bundles structure of the shoot node

**Figure 2:**
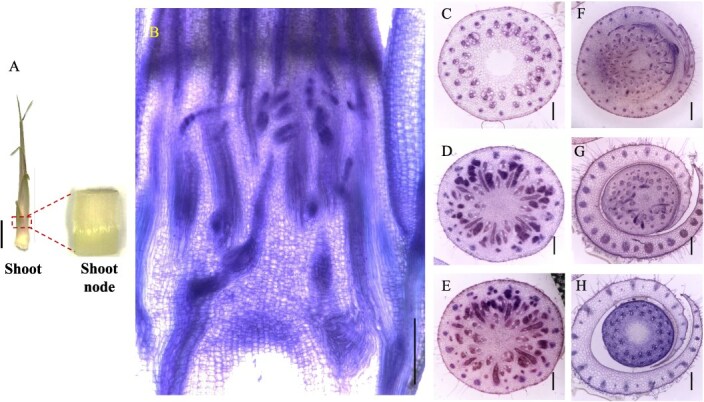
Morphology and vascular systems of the bamboo shoot node. (A) Morphology of the bamboo shoot node. (B) Longitudinal view of vascular systems in the bamboo shoot node. (C–H) Transverse view of vascular systems in the bamboo shoot node from lower to upper in six positions. Transverse and longitudinal sections (100 μm) of Moso bamboo shoot nodes were cut with a microtome and double stained with toluidine blue and safranine. Longitudinal view (B) of the whole node and transverse view (C–H) of different sections from lower to upper nodes were photographed. Scale bar in A is 1 cm, and scale bars in B–H are 300 μm.

In bamboo shoots, nodes are encased by the shoot sheath (Fig. 2A), and each node is connected to the upper and lower nodes via an internode, similar to culm nodes (Fig. 2A). From the longitudinal view, the vascular bundles in the shoot node exhibit a complex arrangement, with both axial and transverse vascular bundles traversing the node (Fig. 2B). At the base of the shoot node, axial vascular bundles predominate, but as the node extends upward, an increasing number of transverse vascular bundles develop (Fig. 2B). In the transverse view (Fig. 2C and D), vascular bundles are differentiate into enlarged vascular bundles, diffuse vascular bundles, and transit vascular bundles. At the top of the node, some vascular bundles differentiate into shoot sheath vascular bundles, which surrounded the node region (Fig. 2G and H). The distribution of transit vascular bundles (large T^L^VBs and small T^S^VBs, yellow and orange in Fig. 8B) remains consistent with that observed in bamboo seedlings. However, the morphology of enlarged vascular bundles (large E^L^VB and small E^S^VBs, red and purple, in Fig. 8B) in the shoot node differs from that in the culm node. In shoot nodes, E^L^VBs exhibit a spoon-like shape, with an underdeveloped phloem region (Fig. 2D and E), whereas in culm nodes, both the xylem and phloem regions are well developed.

### Anatomy and vascular bundles structure of the rhizome node

**Figure 3:**
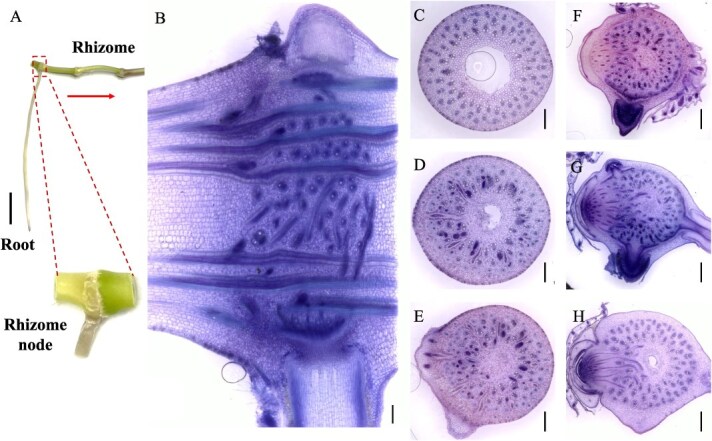
Morphology and vascular systems of the bamboo rhizome node. (A) Morphology of the bamboo rhizome node. (B) Longitudinal view of vascular systems in the bamboo rhizome node. (C–H) Transverse view of vascular systems in the bamboo rhizome node from lower to upper in six positions. Transverse and longitudinal sections (100 μm) of Moso bamboo rhizome nodes were cut with a microtome and double stained with toluidine blue and safranine. Longitudinal view (B) of the whole node and transverse view (C–H) of different sections from lower to upper nodes were photographed. Scale bar in A is 1 cm, and scale bars in B–H are 300 μm. Red arrow means the bamboo rhizome extends horizontally from proximal to distal of the parent plant.

Unlike the vertically growing culm and shoot nodes, the rhizome node exhibits a horizontal distribution as it develops underground. It is connected to adjacent nodes on the left and right through internodes (Fig. 3A). In a longitudinal view, the primary axial vascular bundles are symmetrically arranged on both sides of the rhizome node’s longitudinal section (Fig. 3B). Furthermore, a complex network of transverse vascular bundles interweaves among the axial vascular bundles in a twisted pattern. The longitudinal view also reveals a typical fibrous root system emerging from the node, forming crown roots, alongside a nascent coronal shoot bud primordium (Fig. 3B).

In a transverse view, on the left side of the rhizome node, numerous vascular bundles are arranged in a radial gradient (Fig. 3C). Unlike culm and shoot nodes, the enlarged vascular bundles in the rhizome node are concentrated in a long strip on one side, lacking a well-developed phloem region (Fig. 3D). Other vascular bundles exist as a unified structure in the form of small vascular bundles (Figs 3D and 8C), with no differentiation into sheath vascular bundles (Fig. 8C). Conversely, on the right side of the node, the vascular bundles exhibit an asymmetrical distribution due to the formation and differentiation of roots and shoot buds (Fig. 3F and G). As the shoot bud develops, numerous elongated, strip-like transverse vascular bundles emerge from the pith cavity (Fig. 3H).

### Fe deposition in nodes

**Figure 4:**
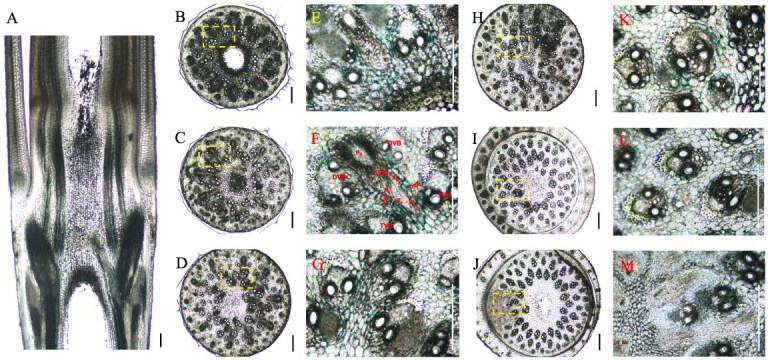
Iron (Fe) deposition in the culm node with Perls’ blue staining. (A) Longitudinal view of Fe deposition in the bamboo culm node. (B–M) Transverse view of Fe deposition in the bamboo culm node. E, F, G, K, L, and M are magnified images of the yellow box areas in B, C, D, H, I, and K, respectively. Transverse and longitudinal sections (100 μm) of Moso bamboo culm nodes were cut with a microtome and stained with Perls’ blue. Longitudinal view (A) of Fe deposition in the whole node and transverse view (B–M) of Fe deposition in different sections from lower to upper nodes were photographed. Blue color shows Fe deposition. Scale bars are 300 μm. EVB, enlarge vascular bundle; DVB, diffuse vascular bundle; TVB, transit vascular bundle; XE, xylem of EVB; PE, phloem of EVB; pcb, parenchyma cell bridge; x, xylem; p, phloem.

Plants require 16 essential elements for normal growth [20], which can be categorized into three groups based on their concentration in various plant tissues: microelements, secondary elements, and macroelement [21, 22]. To investigate elements deposition in nodes, Fe and Zn were selected as representatives of microelements, Ca as a secondary element, and K as a microelement. Their distribution was visualized using different staining approaches techniques. Fe deposition is indicated by blue coloration. In the culm node, Fe accumulation was primarily observed in the cells adjacent to the axial vascular bundles, with intensity gradually decreasing from the bottom to the top of the node (Fig. 4A). In transverse view, Fe tended to accumulate in cells adjacent to both enlarged and small vascular bundles (Fig. 4B–G), whereas minimal Fe deposition was observed in the xylem region of vascular bundles (Fig. 4E and F). Similar patterns were found in in the shoot node, where Fe was predominantly localized in the cells next to axial vascular bundles, with minimal presence in the vascular bundles themselves (Fig. S1B and C). In transverse view, only a small amount of Fe deposition was found in the lower and upper regions of the node. However, Fe accumulation was particularly intense in specific areas, such as cells adjacent to vascular bundles and parenchyma cells (Fig. S1K and L). Compared to culm and shoot nodes, Fe deposition was significantly weaker in the rhizome node (Fig. S5). Both longitudinal and transverse views revealed faint Fe accumulation in cells near the crown root and shoot bud primordia (Fig. S5H and K).

### Zn deposition in nodes

**Figure 5:**
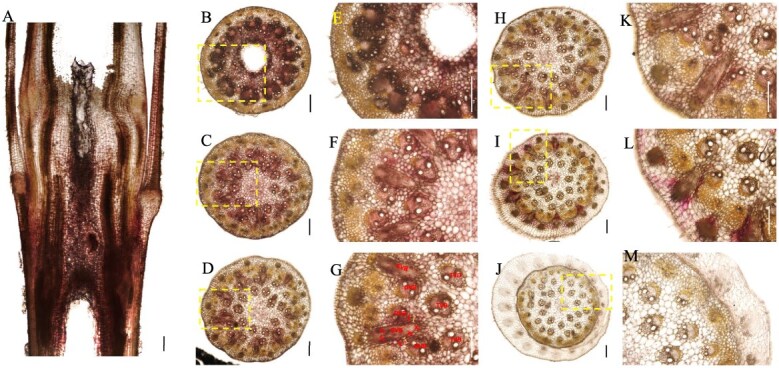
Zinc (Zn) deposition in the culm node with dithizone methanol staining. (A) Longitudinal view of Zn deposition in the bamboo culm node. (B–M) Transverse view of Zn deposition in the bamboo culm node. E, F, G, K, L, and M are magnified images of the yellow box areas in B, C, D, H, I, and K, respectively. Transverse and longitudinal sections (100 μm) of Moso bamboo culm nodes were cut with a microtome and stained with dithizone methanol. Longitudinal view (A) of Zn deposition in the whole node and transverse view (B–M) of Zn deposition in different sections from lower to upper nodes were photographed. Red color shows Zn deposition. Scale bars are 300 μm. EVB, enlarge vascular bundle; DVB, diffuse vascular bundle; TVB, transit vascular bundle; XE, xylem of EVB; PE, phloem of EVB; pcb, parenchyma cell bridge; x, xylem; p, phloem.

Zn deposition is indicated by red coloration. In the longitudinal view of the culm node, Zn accumulation was observed in axial vascular bundles and in parenchyma cells (Fig. 5A), with more intense coloration at the junction between the leaf sheath and the node. In transverse view, Zn deposition was evident in vascular bundles, including both enlarged (E^L^VBs) and small (D^L^VBs, D^S^VBs, and T^L^VBs) vascular bundles in the lower part of the node (Fig. 5B, C, E, and F). As the node extended upward, Zn accumulation in vascular bundles decreased, while stronger deposition was observed in cells located outside the enlarged vascular bundles (Fig. 5H and K). This suggests that Zn may be transported from axial vascular bundles to other cells and tissues through intercellular transfer processes (Fig. 5K and L).

In the shoot node, Zn deposition was predominantly observed in axial vascular bundles, with minimal presence in parenchyma cells (Fig. S2A). In transverse view, red coloration was present in transverse vascular bundles, whereas only faint Zn deposition was detected in the xylem region of small vascular bundles (Fig. S2D, G, H, and K). Compared to culm and shoot nodes, Zn accumulation in the rhizome node was significantly weaker. Both longitudinal and transverse views showed faint Zn deposition in vascular bundles, with no detectable accumulation in crown root cells (Fig. S6).

### Ca deposition in nodes

**Figure 6:**
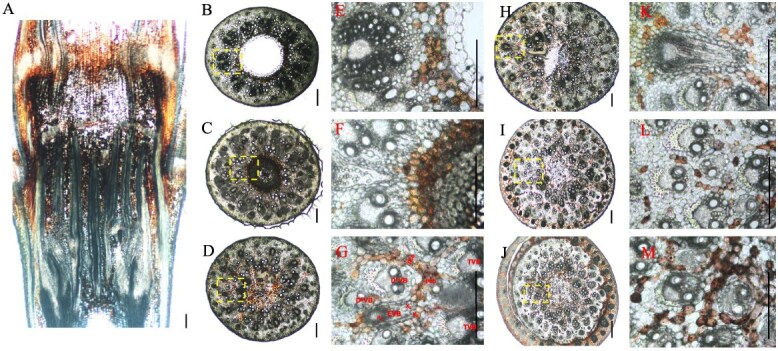
Calcium (Ca) deposition in the culm node with silver nitrate staining. (A) Longitudinal view of Ca deposition in the bamboo culm node. (B–M) Transverse view of Ca deposition in the bamboo culm node. E, F, G, K, L, and M are magnified images of the yellow box areas in B, C, D, H, I, and K, respectively. Transverse and longitudinal sections (100 μm) of Moso bamboo culm nodes were cut with a microtome and stained with silver nitrate. Longitudinal view (A) of Ca deposition in the whole node and transverse view (B–M) of Ca deposition in different sections from lower to upper nodes were photographed. Tan color shows Ca deposition. Scale bars are 300 μm. EVB, enlarge vascular bundle; DVB, diffuse vascular bundle; TVB, transit vascular bundle; XE, xylem of EVB; PE, phloem of EVB; pcb, parenchyma cell bridge; x, xylem; p, phloem.

Ca deposition is indicated by tan coloration. In the culm node, Ca accumulation was lighter at the bottom of the node but increased in intensity as the node extended upward, suggesting that Ca predominantly deposits in the upper regions of the node (Fig. 6A). Minimal Ca was found in enlarged vascular bundles (D^L^VBs) and diffuse vascular bundles (D^L^VBs, D^S^VBs) (Fig. 6A, C, D, F, G, and K), while most Ca accumulation was observed around these vascular bundles (Fig. 6D and G). This pattern is more evident in the magnified figures (Fig. 6G and K), where Ca was detected in the cell layers adjacent to enlarged and diffuse vascular bundles, specifically in diffuse cells, and parenchyma cells. In addition, Ca deposition was observed in the cells surrounding leaf sheath vascular bundles (Fig. 6I and J). Ca accumulation was weak in the xylem region of enlarged vascular bundles (Fig. 6K) but prominent in the surrounding cells, suggesting that Ca probably transported from xylem cells to diffuse and parenchyma cells, where it either accumulates more readily or remains for a longer duration.

**Figure 7:**
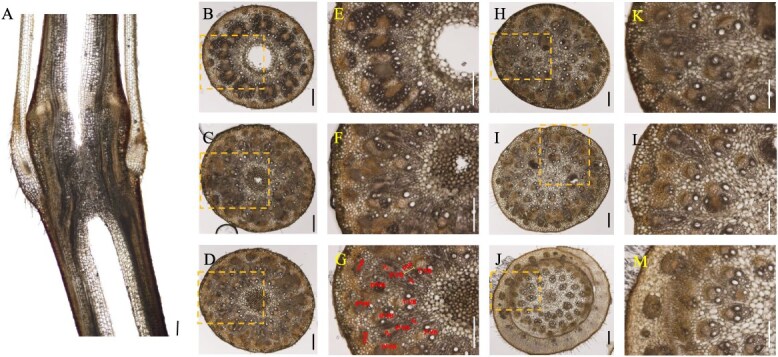
Potassium (K) deposition in the culm node with sodium hexanitrocobaltate staining. (A) Longitudinal view of K deposition in the bamboo culm node. (B–M) Transverse view of K deposition in the bamboo culm node. E, F, G, K, L, and M are magnified images of the yellow box areas in B, C, D, H, I, and K, respectively. Transverse and longitudinal sections (100 μm) of Moso bamboo culm nodes were cut with a microtome and stained with sodium hexanitrocobaltate. Longitudinal view (A) of K deposition in the whole node and transverse view (B–M) of K deposition in different sections from lower to upper nodes were photographed. Yellowish-gray color shows K deposition. Scale bars are 300 μm. EVB, enlarge vascular bundle; DVB, diffuse vascular bundle; TVB, transit vascular bundle; XE, xylem of EVB; PE, phloem of EVB; pcb, parenchyma cell bridge; x, xylem; p, phloem.

In the shoot node, Ca deposition was present in vascular bundles and in the cell layers adjacent to them (Fig. S3A). Besides, Ca accumulation was detected in the bamboo sheath (Fig. S3A). Transverse views further confirmed that Ca was found in both enlarged and small vascular bundles as well as in the surrounding cell layers (Fig. S3C and F). In the rhizome node, Ca deposition was weaker than in the culm and shoot nodes. Tan coloration was observed in the cell layers adjacent to the pith cavity and in the outer region of the node (Fig. S7A, F, and G). A dense accumulation of Ca was also observed in some transverse vascular bundles, as well as in the crown root and shoot bud primordia cells originating from the node (Fig. S7H and I).

### K deposition in nodes

K deposition is indicated by yellowish-gray coloration. As a macroelement, K was found in almost all cells of the culm node; however, variations in K deposition were observed. In the longitudinal view, strong yellowish-gray coloration was detected in vascular bundles and parenchyma cells, while weaker coloration was observed in cells near the pith cavity (Fig. 7A). In the transverse view, K was present in both the xylem and phloem regions of vascular bundles (Fig. 7C, D, F, and G). However, some specific cells, such as pith cavity cells and xylem transfer cells (in the xylem region of E^L^VBs), exhibited no detectable yellowish-gray coloration (Fig. 7F and G). In the shoot node, K deposition was also high in vascular bundles but lower in cells near the pith cavity (Fig. S4B–G). Strong yellowish-gray coloration was observed in the phloem regions of vascular bundles and in the vascular bundles of the leaf sheath (Fig. S4I, J, L, and M). However, in the transverse view, K deposition in the shoot node was notably weaker than in the culm node (Fig. 7B–M and Fig. S4B–M). In the rhizome node, K deposition followed a pattern similar as to that in the culm and shoot nodes. However, a notable difference was observed in the vascular bundles of the coronary shoot and bud primordia, where the yellowish-gray coloration was significantly more intense (Fig. S8H, I, K, and L).

**Figure 8:**
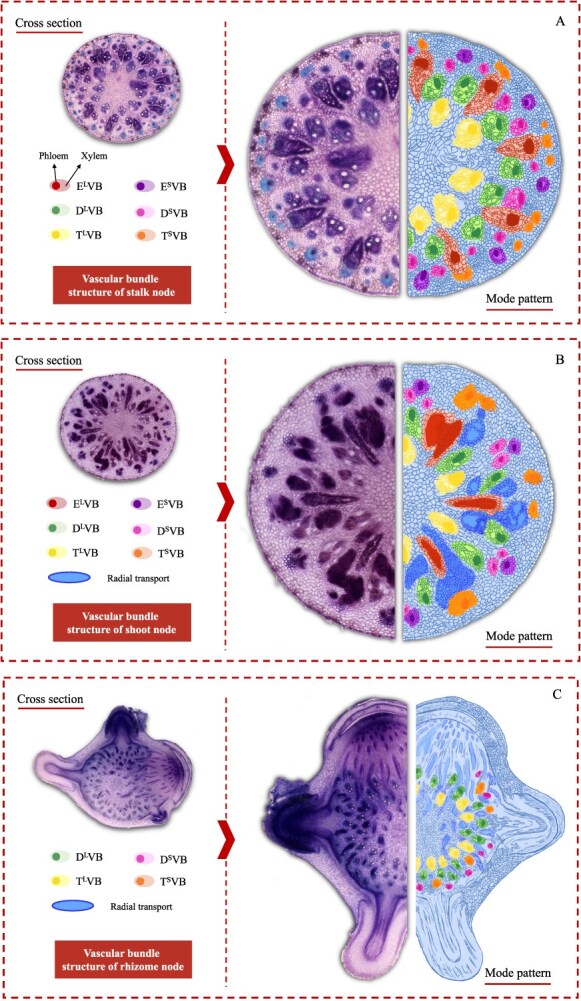
Schematic structures of vascular bundles in bamboo nodes. (A) Schematic structures of vascular bundles in culm node. (B) Schematic structures of vascular bundles in the shoot node. (C) Schematic structures of vascular bundles in the rhizome node. Transverse section (100 μM) of Moso bamboo culm, shoot, and rhizome nodes were cut with a microtome and double stained with toluidine blue and safranine. Three different phases of axial VBs are shown: enlarged VB (EVB), divided into large (E^L^VB; red) and small (E^S^VB; purple); diffuse VB (DVB), divided into large (D^L^VB; green) and small (D^S^VB; pink); and transit VB (TVB), divided into large (T^L^VB; yellow) and small (T^S^VB; orange). Blue color shows the cell wall. Solid and shaded areas within each mark of VBs indicate phloem and xylem regions, respectively.

## Discussion

### Differentiation of vascular bundles in culm, shoot, and rhizome nodes

Our findings reveal a complex yet well-organized vascular system in the nodes of the culm, shoot, and rhizome. These nodes share structural and compositional similarities in their vascular systems, as both axial and transverse vascular bundles traverse the nodes. Their fundamental components—enlarged, diffuse, and transit vascular bundles, along with parenchyma tissue—constitute the basic framework of these nodes (Figs 1–3 and 8). However, notable differences exist among them. In the culm node, the enlarged vascular bundles are well developed and clearly differentiated, featuring distinct xylem and phloem regions (Figs 1 and 8A). In contrast, the shoot and rhizome nodes exhibit less developed enlarged vascular bundles, with an underdeveloped or indistinct phloem region (Figs 2, 3, and 8B and C). This raises the following question: Why is the phloem poorly developed in the shoot and rhizome nodes? The phloem is primarily responsible for transporting soluble organic compounds, such as photo-assimilates [22, 23]. During the shoot stage, the shoot remains enclosed by the shoot sheath, which limited its assimilative capacity, and results in a lower demand for photo-assimilate transport. Consequently, a specialized phloem vascular system is unnecessary in the shoot node, and the transport of these compounds is instead facilitated by smaller vascular bundles with a well-developed phloem system (Fig. 8B). Similarly, the undeveloped phloem in the rhizome node (Fig. 8C) suggests that the demand for soluble organic compounds in the structure is also relatively low. The phloem within smaller vascular bundles appears sufficient to support the basic distribution and transport of these compounds.

Another significant difference was observed in the rhizome node compared to the culm and shoot nodes. Our results indicate that rhizome nodes develop vascular bundles specifically for crown roots and new coronal shoot buds, supporting the emergence of new roots and shoots (Figs 3 and 8B). This suggests that beyond its typical function in nutrient allocation and transport, the rhizome node plays a crucial role in facilitating the exchange of nutrients absorbed from the roots and organic compounds transported from the shoots. Additional structural distinctions were also identified. While culm nodes develop concentric leaf sheath vascular bundles (Fig. 1G and H) and shoot nodes form spiral-patterned shoot sheath bundles (Fig. 2G and H), rhizome nodes completely lack sheath-specific vascular bundles. This differentiation in vascular structure is closely linked to their functional specialization. Culm and shoot nodes serve as junctions connecting leaves and shoot sheaths, necessitating specialized vascular bundles to support these aerial organs. In contrast, rhizome nodes primarily function as underground hubs, linking roots and shoots, making the development of sheath vascular bundles less critical. Consequently, sheath bundle differentiation is absent in rhizome nodes after rhizome formation, reflecting an evolutionary adaptation to their role in resource integration rather than in supporting aerial organs.

### The hub role of nodes in mineral element distribution and transfer in bamboo

Traditionally, it is believed that the allocation and transfer of mineral elements in plants are determined by transpiration [24]. For instance, leaves with a larger surface area typically receive more mineral elements due to higher transpiration rates. However, certain meristematic tissues, such as new leaves, flowers, and growing seeds, require substantial mineral elements for their active growth despite having low transpiration rates [25]. Similarly, in this study, similar observations were made regarding the growth of shoots and rhizomes. The bamboo shoot is enclosed by a sheath, and the rhizome grows underground, both indicating low transpiration conditions during their active growth. How do plants reconcile this paradox?

**Figure 9:**
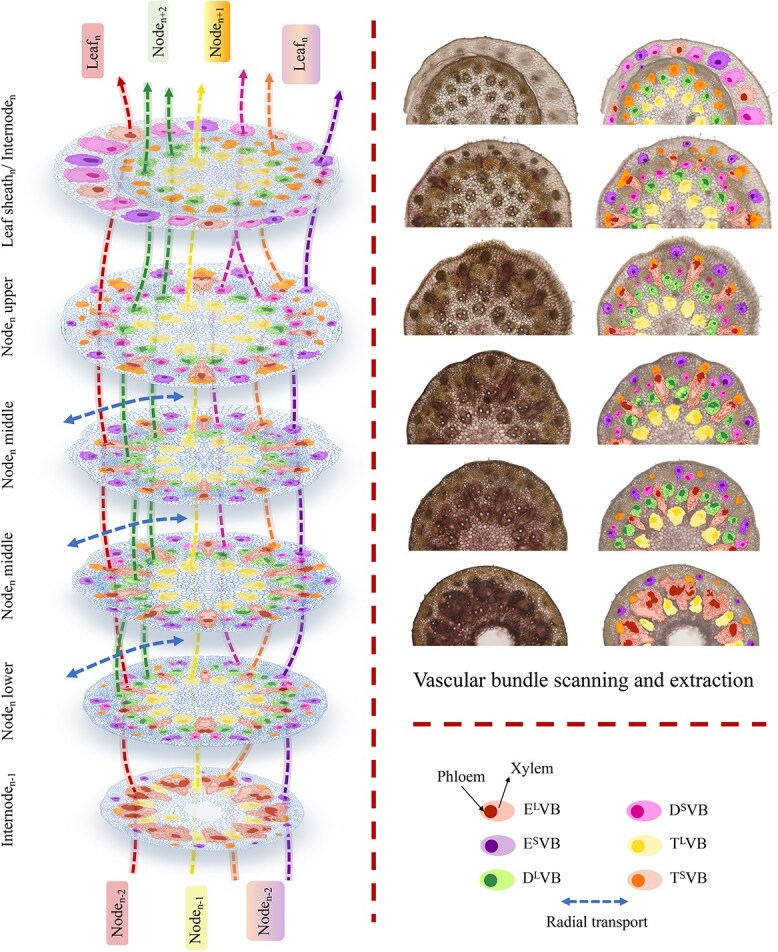
Vascular system in a Moso bamboo culm node. Schematic structures of vascular bundles (VBs) drawn on the pictures. Three different phases of axial VBs are shown: diffuse VB (DVB) divided into large (D^L^VB; green) and small (D^S^VB; pink); enlarged VB (EVB), divided into large (E^L^VB; red) and small (E^S^VB; purple); and transit VB (TVB), divided into large (T^L^VB; yellow) and small (T^S^VB; orange). Radial transport of vascular bundles is shown in blue. Solid and shaded areas within each mark of VBs indicate phloem and xylem regions. Arrows with the same color show the connection of VBs between the sections.

Recent studies have highlighted the pivotal role of nodes in gramineous plants in the distribution of mineral elements [3]. For example, in rice, node I (the uppermost node) regulates the allocation of mineral elements to the flag leaf or panicle. In this study, we examined bamboo, which differs from other gramineous plants in possessing three distinct types of nodes: culm, shoot, and rhizome nodes. The culm node, which shares structural and anatomical similarities with the rice node and connects to the leaves, plays a central role in the distribution of elements above ground. This is supported by the accumulation of Fe, Zn, Ca, and K in different regions of the culm node. Our findings indicate that mineral elements exhibit distinct deposition patterns within nodes. For example, Fe accumulates predominantly in cells adjacent to both enlarged and small vascular bundles, while Zn is mainly localized in the xylem regions of both these vascular bundle types (Figs 4 and 5). Conversely, K is distributed ubiquitously across all node cells (Fig. 7), whereas Ca is preferentially found in cells surrounding both small and enlarged vascular bundles (Fig. 6). These results suggest that the distribution of mineral nutrients varies depending on the specific element. Taking Zn as an example, we combined different vascular bundle pathways to simulate the transport of zinc in the culm node. Zn was primarily deposited along the boundaries of E^L^VBs in the lower region of the node (Figs 5A and B and 9). As it moved upward, Zn was increasingly detected in E^L^VBs, D^L^VBs, D^S^VBs, and T^L^VBs (Figs 5C, D, F, and G), suggesting that Zn is gathered and allocated to specialized vascular bundles (Figs 5D–F and 9). In the upper region of the node, Zn accumulated at the junction between D^L^VBs and D^S^VBs, while the mature leaf sheath exhibited an alternating structure with D^L^VB and D^S^VB, facilitating Zn transfer from the node to the leaf sheath and ultimately to the target leaf (Figs 5H, I, K, and L and 9). Additionally, a portion of Zn was transported to the upper node via D^L^VBs, D^S^VBs, and T^L^VBs (Fig. 9). This adaptive mechanism ensures the proper allocation of Zn according to changing conditions in bamboo and prioritizes the high Zn demands of actively growing tissues located farther from the transpiration site.

In contrast, the structure and anatomy of shoot and rhizome nodes differ slightly from those of culm nodes (Fig. 8). Do these nodes also play a central role in mineral element allocation? Based on structural and anatomical analyses, we found that shoot and rhizome nodes possess a complex but well-organized vascular bundle system, characterized by enlarged vascular bundles in the node region, indicating their critical role in mineral element allocation. Although the staining intensity of different elements was weaker in shoot and rhizome nodes than in culm nodes, these nodes remain key sites for mineral element distribution. Given that nodes serve as hubs for mineral element allocation, an important question arises: what mechanisms regulate this process? In rice, specific transporters such as OsFRDL1, OsZIP3, OsSPDT, and OsLsi6 have been identified as key regulators of Fe, Zn, P, and Si distribution in nodes [4]. However, no transporters have yet been identified or functionally characterized in bamboo nodes. Future research is needed to identify and functionally characterize the transporters responsible for the specific distribution of elements in different bamboo nodes.

In conclusion, despite their structural and anatomical differences, the culm, shoot, and rhizome nodes share functionally similar vascular bundle systems. By linking the structure of vascular bundles with the spatial deposition of mineral elements in various node regions, we reveal the hub role of nodes in mineral element distribution in bamboo.

## Materials and methods

### Plant materials and growth conditions

Initially, Moso bamboo seeds were soaked in deionized water for 2 days at 25°C in a dark incubator. Subsequently, the seeds were moved to a Petri dish with humid filter paper for a period of 7 days to facilitate germination. After germination, the seedlings were moved on a black net, which was floating on 0.5 mM CaCl_2_ solution for 1 week. Then the seedlings were transplanted into a 3-l black plastic pot containing a half strength Kimura B nutrient solution (pH = 5.6), with the specific element composition in the nutrient solution as described previously [26]. The nutrient solution was replaced every 3 days. The plants were cultivated in a glass greenhouse under natural light, at a temperature of 25°C and a humidity level of 70%.

For culm nodes collection, the seedlings prepared above were cultivated in nutrient solution until use (60 days old seedlings). Then the nodes in the culm with leaf sheath were cut with a razor and kept in a 15-ml centrifuge tube with distilled water for using.

For shoot nodes collection, the seedlings prepared above were cultivated in nutrient solution until the shoot appeared (45 days old seedlings). Then the shoots were harvested, and the outermost two layers of bamboo shoot sheaths were removed. The nodes in the shoots were cut with a razor and kept in a 15-ml centrifuge tube with distilled water for using.

For rhizome nodes collection, the seedlings prepared above were cultivated in nutrient solution until the rhizome appeared (142 days old seedlings). Then the nodes with roots in the rhizome were cut with a razor and kept in a 15-ml centrifuge tube with distilled water for using.

### Bamboo node slice preparation

Transverse and longitudinal sections (100 μM) of Moso bamboo nodes were cut with a microtome (Leica RM2265, Wetzlar, Germany) at room temperature and transferred onto glass slides in distilled water until staining use. All the transverse sections were sequentially cut from a single node, and to guarantee reproducibility, triplicate biological replicates were employed using separate and independent nodes.

### Staining of vascular system in the node

The vascular system of the node in the culm, shoot, and rhizome was double stained with toluidine blue and safranine (CI 52040, Serva, Germany) according to Ref. [27]. Briefly, the bamboo node slice was stained with toluidine blue (0.05%, w/v) for 5 min after washing with distilled water. The node slice was stained with safranine (0.1%, w/v) for 1 min after washing with ethanol (70%). Then the bamboo slice was observed and photographed with a stereoscopic microscope (Discovery.V12, ZEISS).

### Staining of iron (Fe), zinc (Zn), calcium (Ca), and potassium (K) in the node

Perls’ blue staining was used for Fe staining. Briefly, the bamboo node slice (100 μM) was exposed to a staining solution, which was mixed with HCl (4%, v/v) and potassium ferrocyanide (4%, w/v) immediately prior to use for 15 min, then the slice was rinsed with distilled water three times and observed and photographed with a fluorescence microscope (Axio Imager.2, ZEISS).

For Zn staining, the bamboo node slice was soaked in dithizone methanol (Aladdin Shanghai) solution (500 mg/l) for 0.5 h, followed by washing three times with distilled water; the slice was observed and photographed with a fluorescence microscope (Axio Imager.2, ZEISS).

For Ca staining, the bamboo node slice was soaked in silver nitrate (SH Test Shanghai) solution (5%) for 1 h in front of a 60-W lamp, then the slice was rinsed three times with distilled water. Then the bamboo node slice was soaked in Hypo (5%) for 5 minutes. After being washed three times, the slice was observed and photographed with a fluorescence microscope (Axio Imager.2, ZEISS).

For K staining, the bamboo node slice was soaked in sodium hexanitrocobaltate (III) (Macklin Shanghai) 0.5 M in 10% (v/v) acetic acid for 10 minutes and rinsed for 1 min in ice-cold deionized water. The slice was observed and photographed with a fluorescence microscope (Axio Imager.2, ZEISS).

## Supplementary Material

Web_Material_uhaf113

## Data Availability

All data supporting the conclusions of this study are present in the paper or the supplementary data.
