# Supplementary material for: Linking the structure of vascular bundles and mineral element deposition reveals the hub role of nodes in bamboo
Source: Hortic Res. 2025 Apr 24;12(8):uhaf113. doi: 10.1093/hr/uhaf113 (PMC12247518; doi:10.1093/hr/uhaf113)
Supplement: Web_Material_uhaf113 [file web_material_uhaf113.zip › Supplementary figures.pptx]

## Slide 1
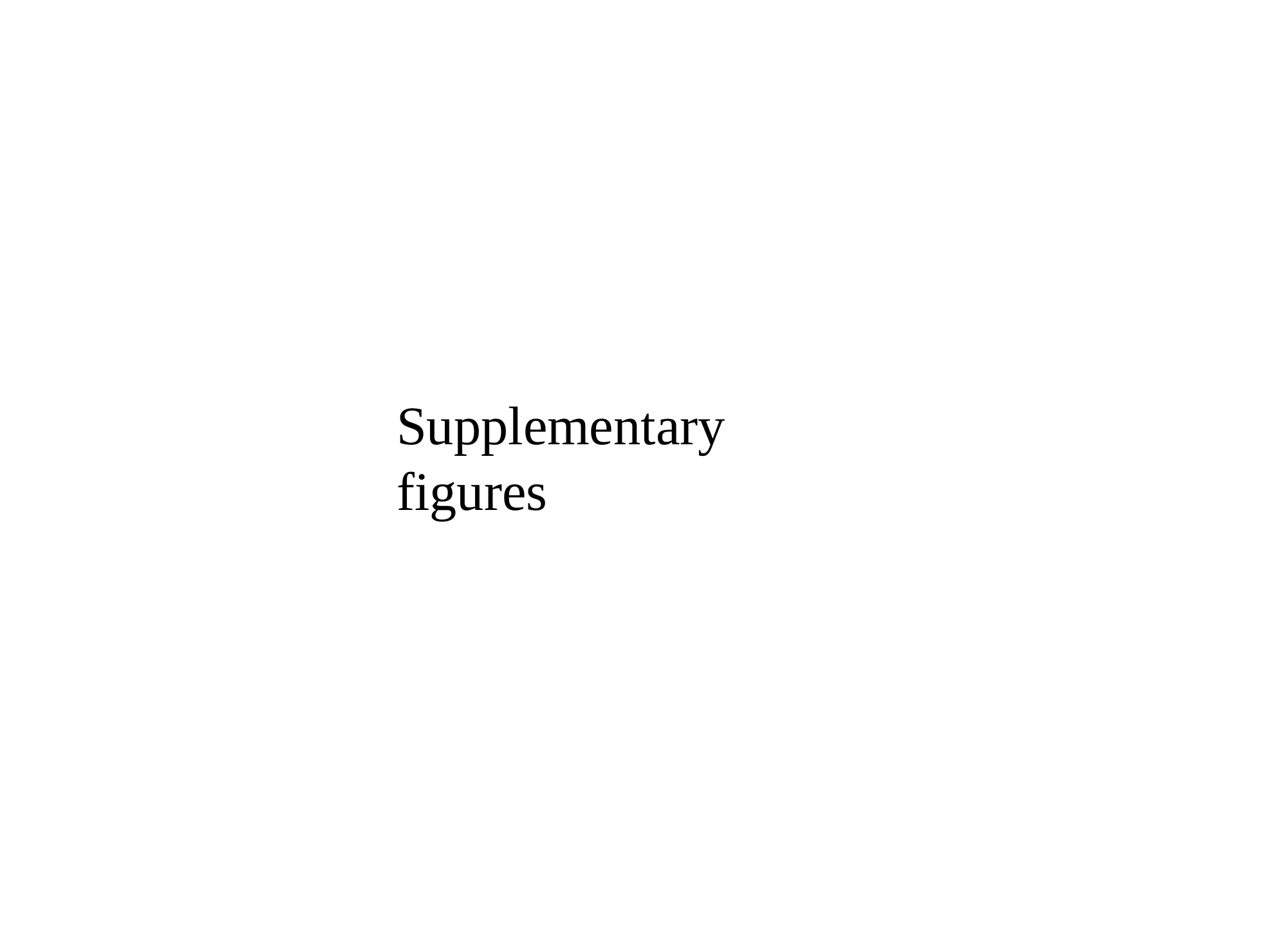

Supplementary figures

## Slide 2
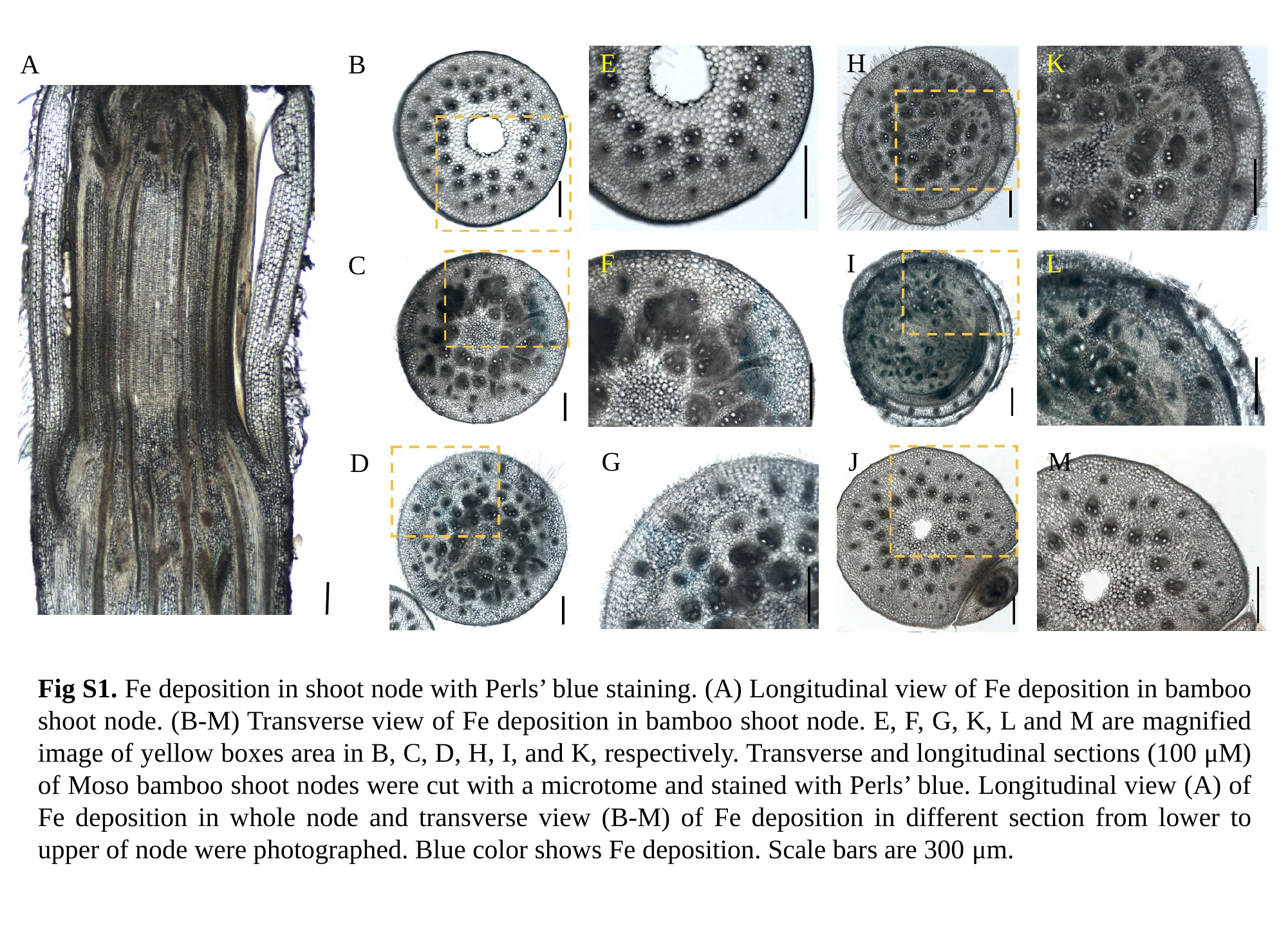

E
H
K
A
B
F
I
L
C
G
J
M
D
Fig S1. Fe deposition in shoot node with Perls’ blue staining. (A) Longitudinal view of Fe deposition in bamboo shoot node. (B-M) Transverse view of Fe deposition in bamboo shoot node. E, F, G, K, L and M are magnified image of yellow boxes area in B, C, D, H, I, and K, respectively. Transverse and longitudinal sections (100 μM) of Moso bamboo shoot nodes were cut with a microtome and stained with Perls’ blue. Longitudinal view (A) of Fe deposition in whole node and transverse view (B-M) of Fe deposition in different section from lower to upper of node were photographed. Blue color shows Fe deposition. Scale bars are 300 μm.

## Slide 3
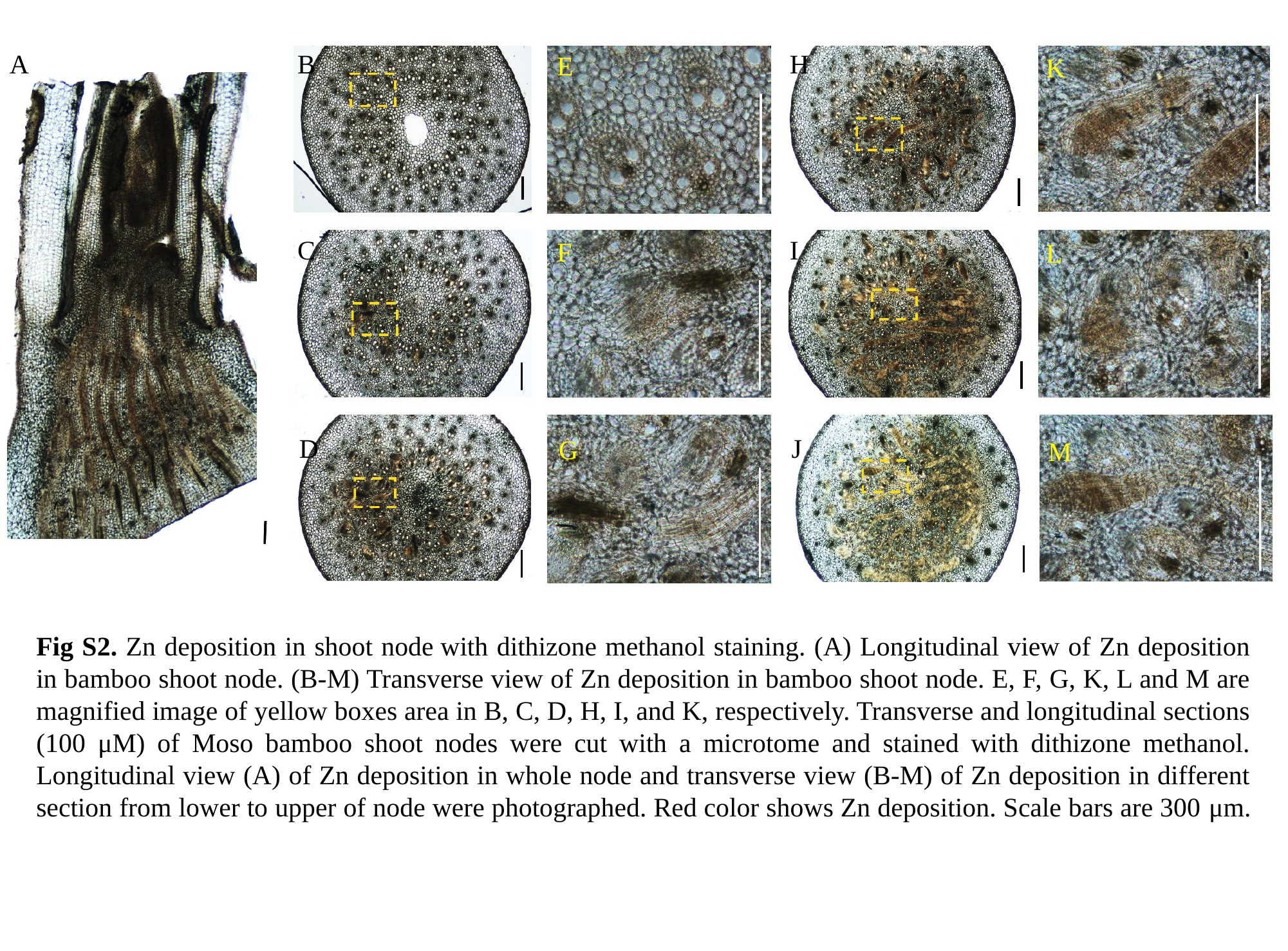

A
B
H
E
K
C
I
F
L
D
J
G
M
Fig S2. Zn deposition in shoot node with dithizone methanol staining. (A) Longitudinal view of Zn deposition in bamboo shoot node. (B-M) Transverse view of Zn deposition in bamboo shoot node. E, F, G, K, L and M are magnified image of yellow boxes area in B, C, D, H, I, and K, respectively. Transverse and longitudinal sections (100 μM) of Moso bamboo shoot nodes were cut with a microtome and stained with dithizone methanol. Longitudinal view (A) of Zn deposition in whole node and transverse view (B-M) of Zn deposition in different section from lower to upper of node were photographed. Red color shows Zn deposition. Scale bars are 300 μm.

## Slide 4
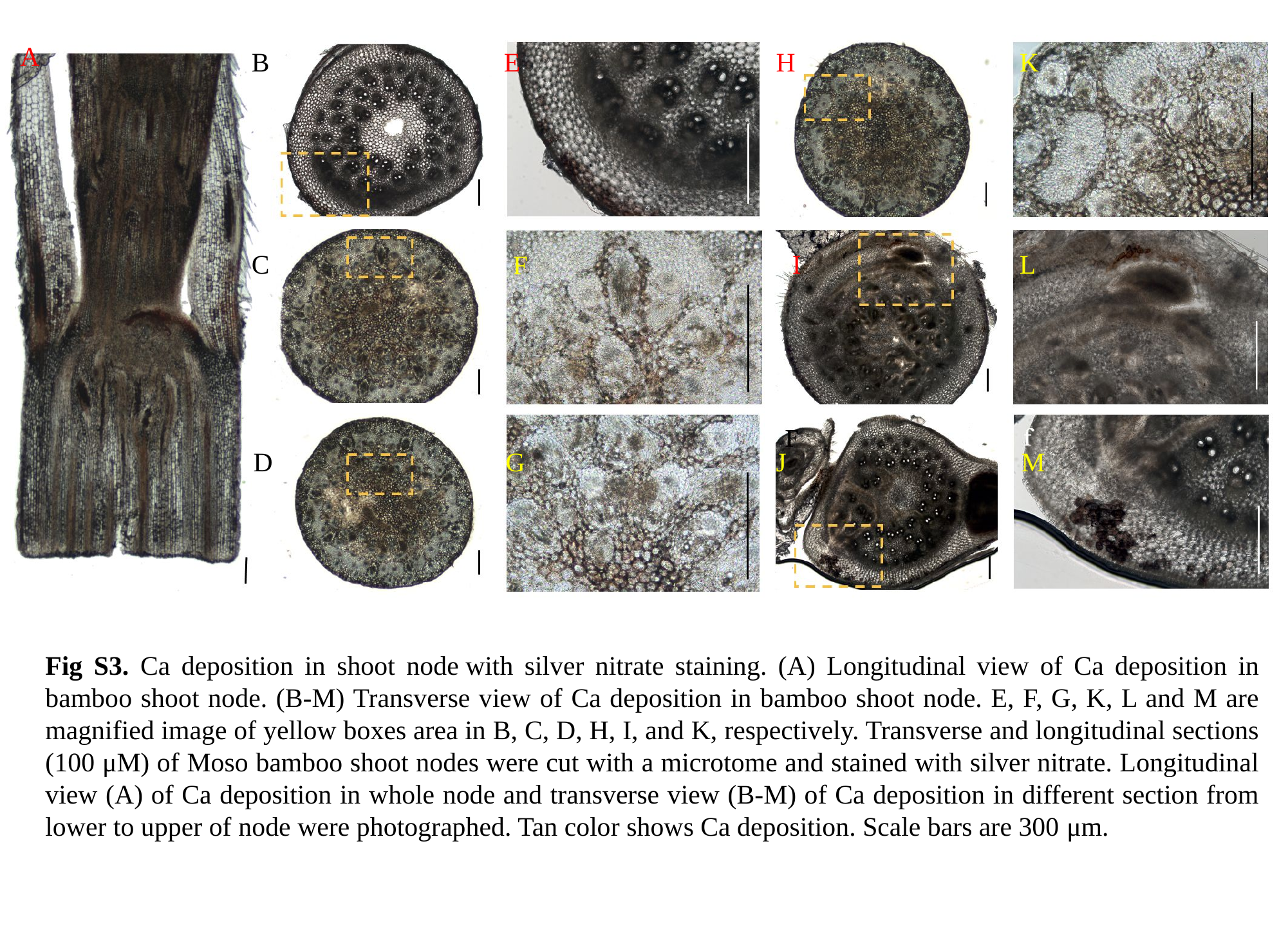

A
H
B
E
K
C
I
L
F
D
G
J
M
Fig S3. Ca deposition in shoot node with silver nitrate staining. (A) Longitudinal view of Ca deposition in bamboo shoot node. (B-M) Transverse view of Ca deposition in bamboo shoot node. E, F, G, K, L and M are magnified image of yellow boxes area in B, C, D, H, I, and K, respectively. Transverse and longitudinal sections (100 μM) of Moso bamboo shoot nodes were cut with a microtome and stained with silver nitrate. Longitudinal view (A) of Ca deposition in whole node and transverse view (B-M) of Ca deposition in different section from lower to upper of node were photographed. Tan color shows Ca deposition. Scale bars are 300 μm.

## Slide 5
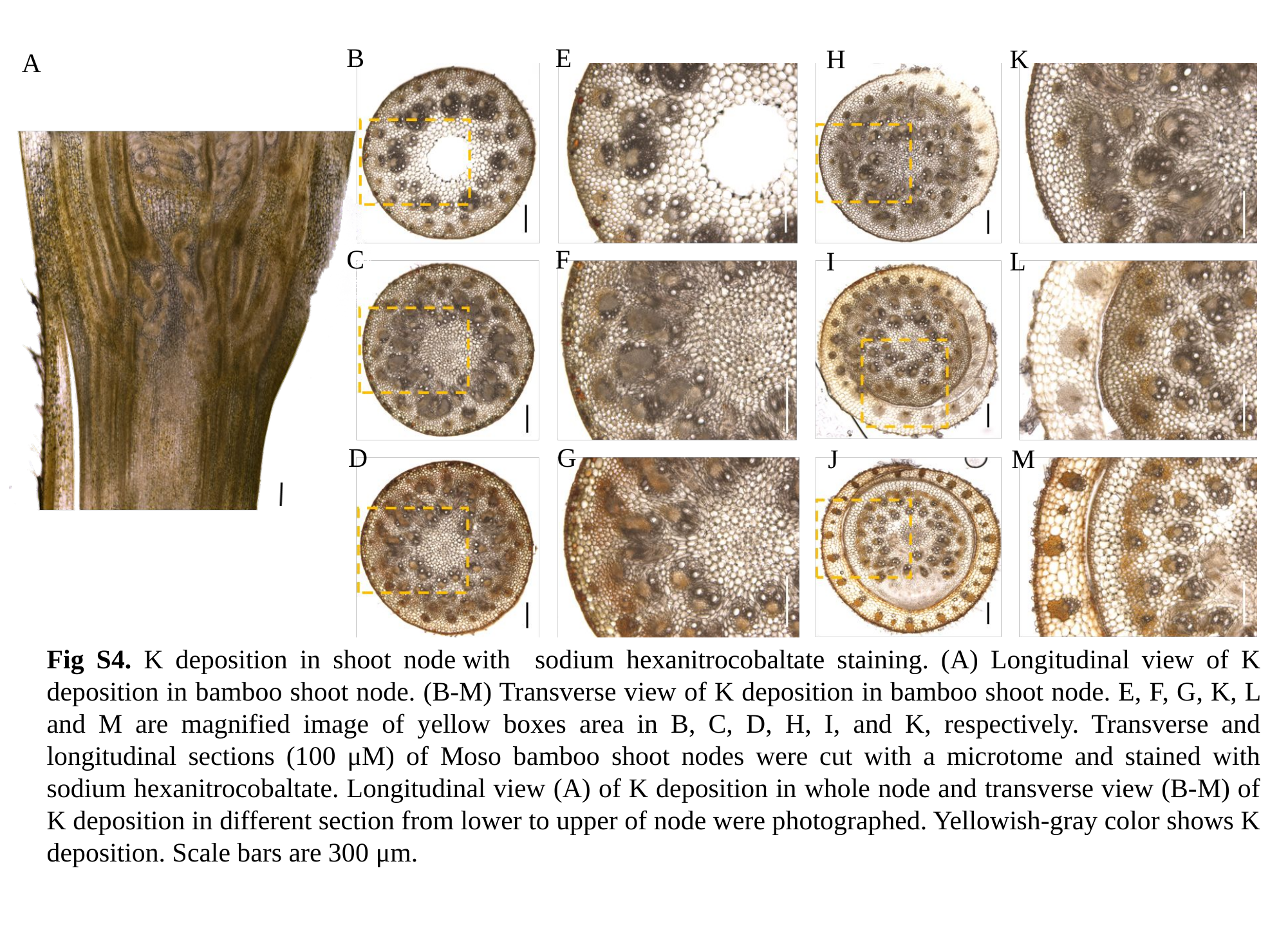

B
E
H
K
A
C
F
I
L
D
G
J
M
Fig S4. K deposition in shoot node with sodium hexanitrocobaltate staining. (A) Longitudinal view of K deposition in bamboo shoot node. (B-M) Transverse view of K deposition in bamboo shoot node. E, F, G, K, L and M are magnified image of yellow boxes area in B, C, D, H, I, and K, respectively. Transverse and longitudinal sections (100 μM) of Moso bamboo shoot nodes were cut with a microtome and stained with sodium hexanitrocobaltate. Longitudinal view (A) of K deposition in whole node and transverse view (B-M) of K deposition in different section from lower to upper of node were photographed. Yellowish-gray color shows K deposition. Scale bars are 300 μm.

## Slide 6
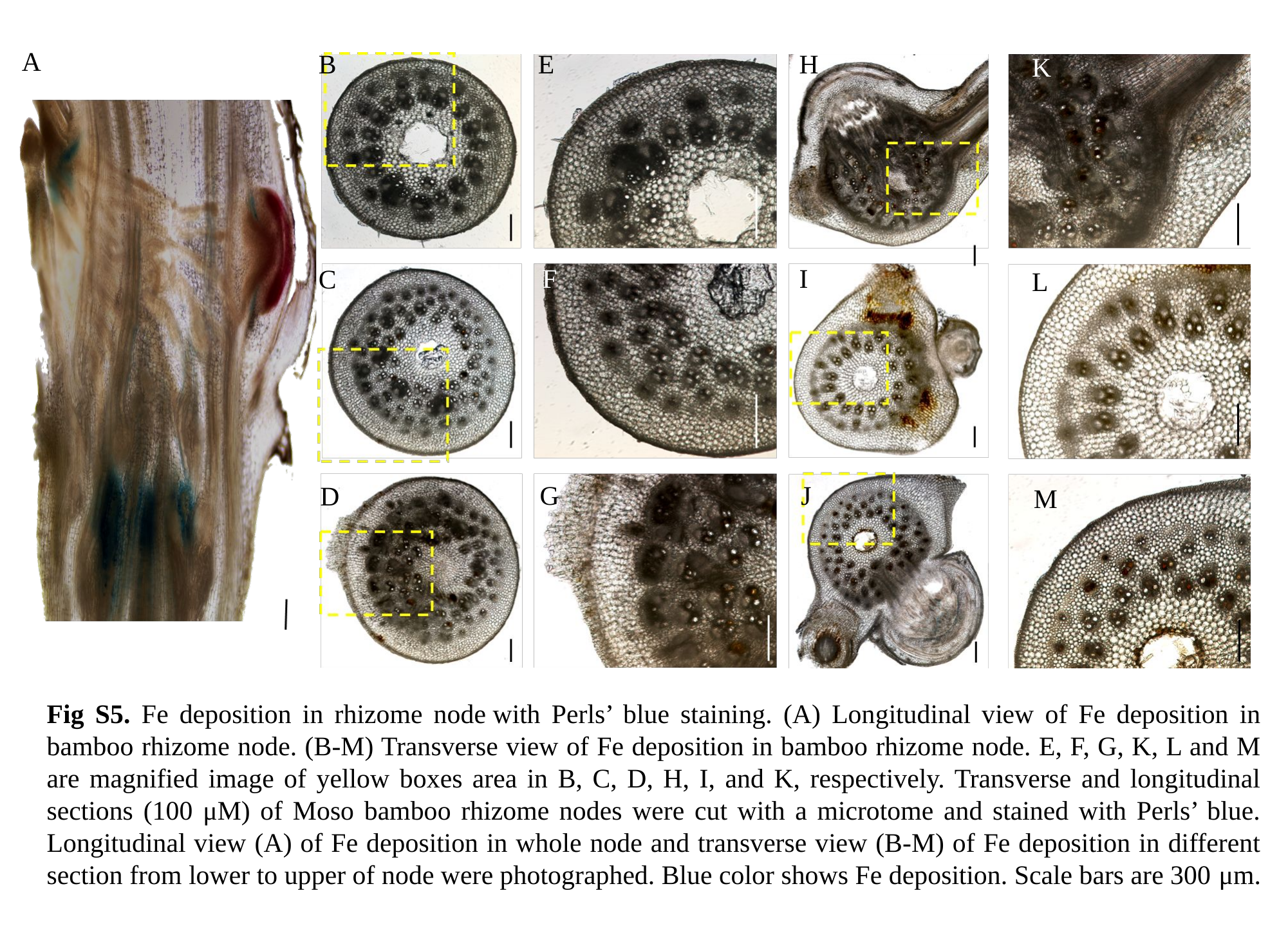

A
E
H
B
K
F
I
C
L
G
J
D
M
Fig S5. Fe deposition in rhizome node with Perls’ blue staining. (A) Longitudinal view of Fe deposition in bamboo rhizome node. (B-M) Transverse view of Fe deposition in bamboo rhizome node. E, F, G, K, L and M are magnified image of yellow boxes area in B, C, D, H, I, and K, respectively. Transverse and longitudinal sections (100 μM) of Moso bamboo rhizome nodes were cut with a microtome and stained with Perls’ blue. Longitudinal view (A) of Fe deposition in whole node and transverse view (B-M) of Fe deposition in different section from lower to upper of node were photographed. Blue color shows Fe deposition. Scale bars are 300 μm.

## Slide 7
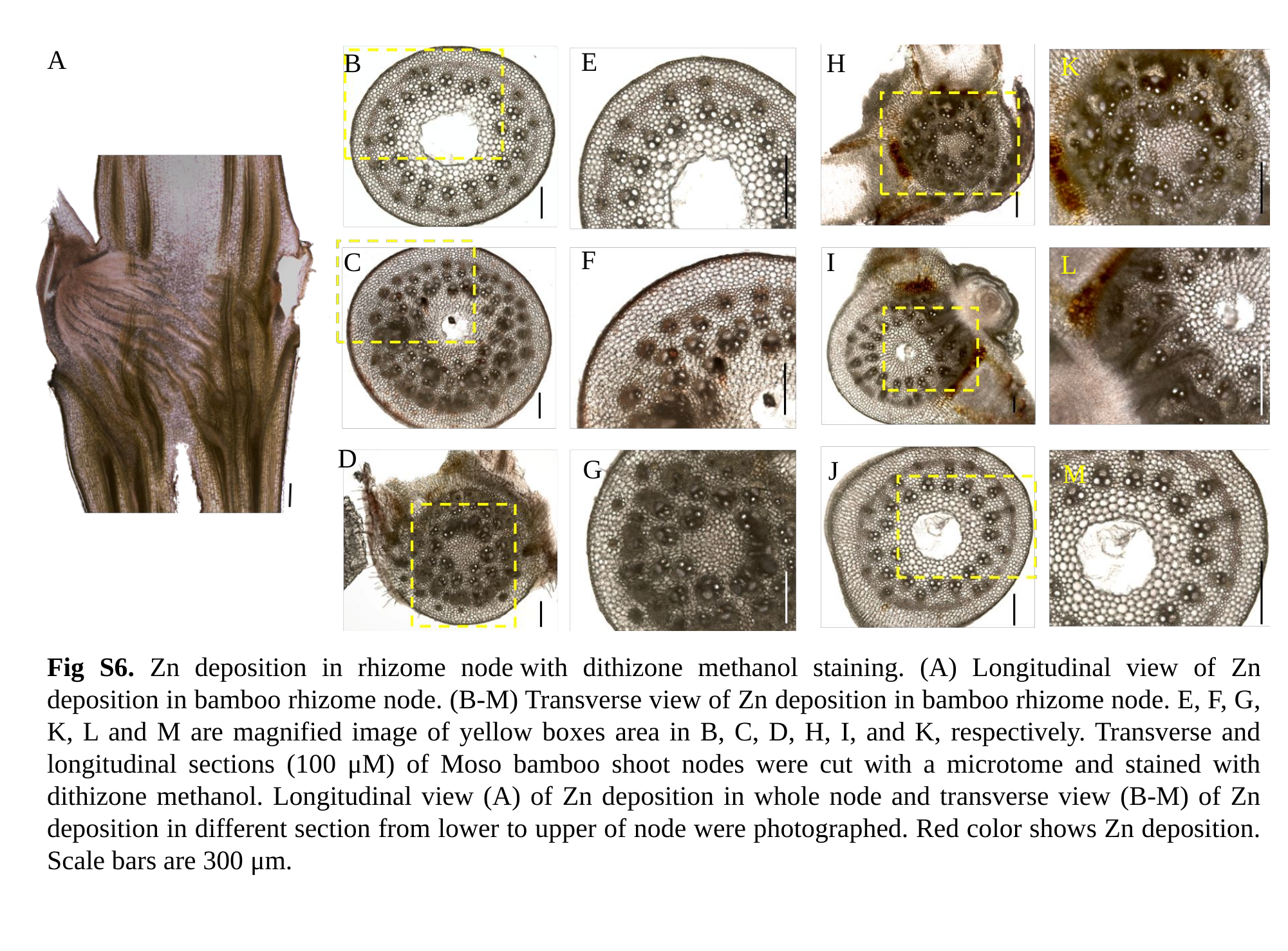

A
E
B
H
K
F
C
I
L
D
G
J
M
Fig S6. Zn deposition in rhizome node with dithizone methanol staining. (A) Longitudinal view of Zn deposition in bamboo rhizome node. (B-M) Transverse view of Zn deposition in bamboo rhizome node. E, F, G, K, L and M are magnified image of yellow boxes area in B, C, D, H, I, and K, respectively. Transverse and longitudinal sections (100 μM) of Moso bamboo shoot nodes were cut with a microtome and stained with dithizone methanol. Longitudinal view (A) of Zn deposition in whole node and transverse view (B-M) of Zn deposition in different section from lower to upper of node were photographed. Red color shows Zn deposition. Scale bars are 300 μm.

## Slide 8
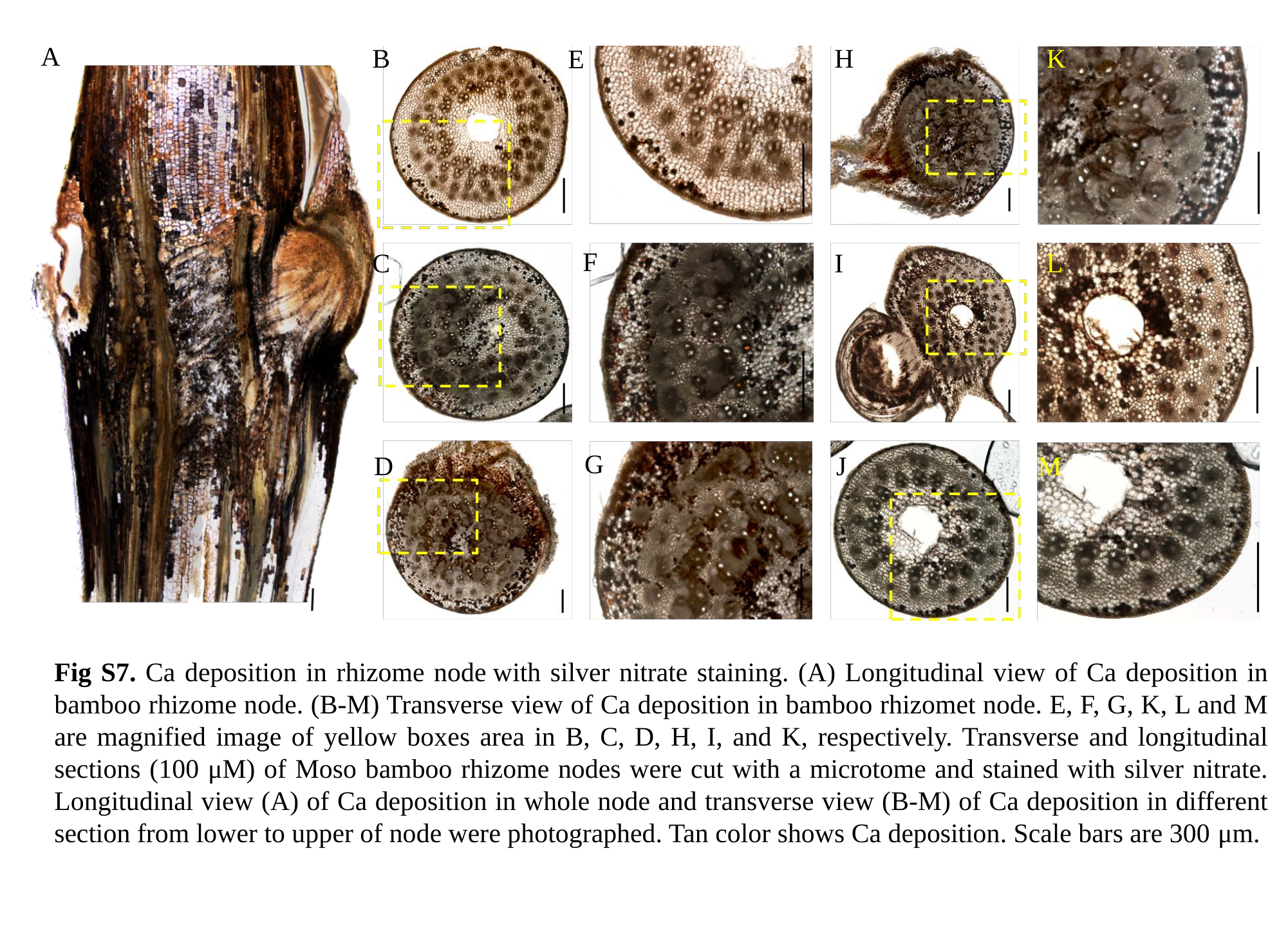

A
H
K
B
E
F
I
L
C
G
J
M
D
Fig S7. Ca deposition in rhizome node with silver nitrate staining. (A) Longitudinal view of Ca deposition in bamboo rhizome node. (B-M) Transverse view of Ca deposition in bamboo rhizomet node. E, F, G, K, L and M are magnified image of yellow boxes area in B, C, D, H, I, and K, respectively. Transverse and longitudinal sections (100 μM) of Moso bamboo rhizome nodes were cut with a microtome and stained with silver nitrate. Longitudinal view (A) of Ca deposition in whole node and transverse view (B-M) of Ca deposition in different section from lower to upper of node were photographed. Tan color shows Ca deposition. Scale bars are 300 μm.

## Slide 9
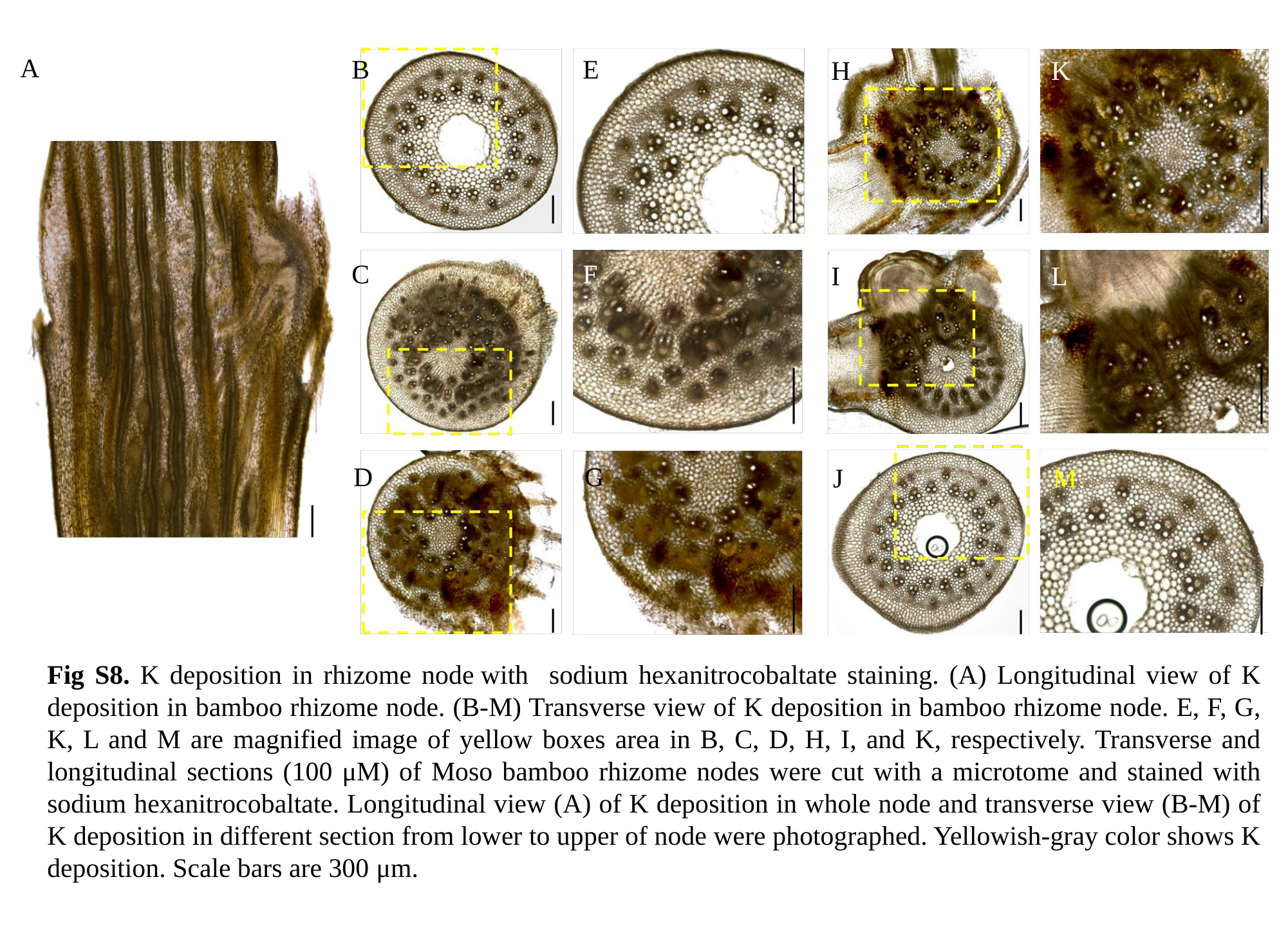

A
B
E
H
K
C
F
I
L
D
G
J
M
Fig S8. K deposition in rhizome node with sodium hexanitrocobaltate staining. (A) Longitudinal view of K deposition in bamboo rhizome node. (B-M) Transverse view of K deposition in bamboo rhizome node. E, F, G, K, L and M are magnified image of yellow boxes area in B, C, D, H, I, and K, respectively. Transverse and longitudinal sections (100 μM) of Moso bamboo rhizome nodes were cut with a microtome and stained with sodium hexanitrocobaltate. Longitudinal view (A) of K deposition in whole node and transverse view (B-M) of K deposition in different section from lower to upper of node were photographed. Yellowish-gray color shows K deposition. Scale bars are 300 μm.
